# Supplementary material for: Subgrouping a Large U.S. Sample of Patients with Fibromyalgia Using the Fibromyalgia Impact Questionnaire-Revised
Source: Int J Environ Res Public Health. 2020 Dec 31;18(1):247. doi: 10.3390/ijerph18010247 (PMC7796452; doi:10.3390/ijerph18010247)
Supplement: Supplementary file 1 [file ijerph-18-00247-s001.zip › Supplementary table 1.docx]

**Supplementary table 1.** Model fit for the 2-5 class LPA solutions

|  | Classes | | | |
| --- | --- | --- | --- | --- |
|  | **2** | **3** | **4** | **5** |
| Free parameters | 64 | 86 | 108 | 130 |
| LL | -295,099.79 | -288,503.66 | -285,944.76 | -284,495.56 |
| BIC | 590,759.27 | 577,759.39 | 572,834.00 | 570,127.99 |
| sBIC | 590,555.90 | 577,486.11 | 572,490.80 | 569,714.89 |
| LMR-LRT *p*-value | < 0.001 | < 0.001 | < 0.001 | 0.111 |
| BLRT *p-*value | < 0.001 | < 0.001 | < 0.001 | < 0.001 |
| Entropy | 0.942 | 0.917 | 0.898 | 0.888 |

***Note:*** LL = log-likelihood; BIC= Bayesian Information Criterion; sBIC = Sample-size-adjusted Bayesian Information Criterion; LMR-LRT = Lo-Mendell-Rubin likelihood ratio test; BLRT= bootstrap likelihood ratio test.
